# Enhanced Tropism of Species B1 Adenoviral-Based Vectors for Primary Human Airway Epithelial Cells

**DOI:** 10.1016/j.omtm.2019.07.001

**Published:** 2019-07-12

**Authors:** Ni Li, Ashley L. Cooney, Wenli Zhang, Anja Ehrhardt, Patrick L. Sinn

**Affiliations:** 1 Department of Pediatrics, The University of Iowa, Iowa City, IA 52242, USA; 2 Institute of Virology and Microbiology, Department of Human Medicine, Faculty of Health, Center for Biomedical Education and Research (ZBAF), Witten/Herdecke University, 58453 Witten, Germany

**Keywords:** coxsackie-adenovirus receptor, CD46, desmoglein 2, gene therapy, respiratory system, lung gene transfer

## Abstract

Adenoviruses are efficient vehicles for transducing airway epithelial cells. Human adenoviruses (Ads) are classified into seven species termed A–G. Most species use the coxsackie-adenovirus receptor (CAR) as a primary cellular receptor. Ad group B is notable because it is further divided into groups B1 and B2 and its members use CD46 or desmoglein 2 (DSG2) as cellular receptors. To date, human Ad types 2 and 5 have been the predominant choices for preclinical and clinical trials using Ad-based viral vectors in the airways. In this study, we screened 14 Ad types representing species C, B1, B2, D, and E. Using well-differentiated primary cultures of human airway epithelial cells (HAEs), we examined transduction efficiency. Based on GFP or nanoluciferase expression, multiple Ad types transduced HAEs as well as or better than Ad5. Ad3, Ad21, and Ad14 belong to species B and had notable transduction properties. We further examined the transduction properties of conditionally reprogrammed airway basal cells and primary basal cells from human lung donors. Again, the transduction efficiency of species B members outperformed the other types. These data suggest that adenoviral vectors based on species B transduce fully differentiated epithelial cells and progenitor cells in the human airways better than Ad5.

## Introduction

Adenoviruses are a diverse family of double-stranded DNA viruses that are associated with a variety of human diseases. To date, over 90 human adenovirus (Ad) genotype numbers have been assigned and sorted into seven species (A–G) (http://hadvwg.gmu.edu/). Ad-based viral vectors are rendered replication incompetent by deleting virally encoded genes and complementing the deletions in producer cells in *trans*. The extent of viral genome deletion ranges from the E1 region alone (first generation) to complete deletion of all virally encoded proteins (helper-dependent Ad [HDAd]). The majority of Ad vectors currently used in preclinical or clinical applications is derived from species C, specifically type 2 (HAdV-C2) or type 5 (HAdV-C5). For brevity, the human adenovirus nomenclature is abbreviated for each Ad-based vector; for example, vectors derived from HAdV-C5 are referred to as Ad5. Viral vectors derived from species C efficiently transduce many airway epithelial cell types, including ciliated, nonciliated, basal, goblet, and submucosal gland cells.^1^, ^2^ However, there may be better choices for airway gene transfer. Biomedical research has explored only a small fraction of Ads, and improvements in Ad taxonomy and genome availability provide an opportunity to explore diverse Ad types as gene delivery vectors.^3^

Efficient transduction of airway epithelial cells is vital for either gene addition or gene editing to be an effective strategy for gene therapy for airway disease such as cystic fibrosis (CF). Gene addition refers to the cellular delivery of a Cystic Fibrosis Transmembrane Conductance Receptor (*CFTR*) expression vector driven by a heterologous promoter. Persistent Ad-meditated gene addition can be achieved using *piggyBac* or Sleeping Beauty hybrid transposon systems.^2^, ^4^, ^5^ Gene editing refers to *in situ* repair of the disease-causing *CFTR* mutation. Ad vectors easily accommodate CRISPR-Cas9, and they are proven delivery vehicles for somatic cell gene editing *in vivo*.^6^, ^7^ For either gene addition or gene editing, stable expression of a functional copy of the *CFTR* gene in the airways would result in an immense improvement in the quality of life of people with CF*.*

In the 1990s, nine CF clinical trials were performed using either Ad2 or Ad5 as the delivery vehicle (reviewed in^55^, ^56^). In general, these studies suggested that Ad-based vectors could partially correct the Cl^−^ transport defect in CF airway epithelia; however, the effects were transient and inflammatory responses were observed. Since that time, the cellular receptor was discovered to be the basolaterally localized coxsackie B virus and adenovirus receptor (CXADR; also known as coxsackie-adenovirus receptor [CAR]),^8^, ^9^ and strategies to dramatically improve Ad-based gene transfer efficiency by transiently opening tight junctions have been standardized.^10^, ^11^ The same strategies that allow access to basolateral receptors also allow access to epithelial cells that are not in direct contact with the lumen, such as basal cells.

The primary receptor for most Ads, including species C and D, is CAR. CAR localizes to the basolateral surface of columnar airway epithelial cells and is a member of the junctional complex that mediates cell-cell adhesion.^9^ Unlike most Ad species, species B viruses bind to different primary cell surface receptors, CD46 or desmoglein 2 (DSG2).^12^, ^13^, ^14^ Similar to CAR, DSG2 localizes to epithelial junctions of stratified epithelial sheets,^15^ including airway, intestines, and urinary tracts. DSG2 is a member of the cadherins family and contributes to cell-cell adhesion.^15^ DSG2 serves as an attachment receptor for entry and lateral spread of species B Ad3 and Ad14.^16^ Interaction between Ad and DSG2 triggers an epithelial-mesenchymal transition that opens intercellular tight junctions to improve access to basolateral receptors.^14^ CD46 is ubiquitously expressed in nearly all cell types except erythrocytes.^17^ Certain species B Ads bind CD46 and enter via an endocytic pathway in epithelial and hematopoietic cells.^18^

Target cells are an important consideration for gene therapy in the airways. Compelling evidence from both *in vitro* and *in vivo* studies indicate that basal cells are multipotent proximal airway progenitor cells that repopulate pulmonary epithelia under normal conditions and during regeneration (reviewed previously^19^, ^20^, ^21^). Cell-labeling experiments with transgenic mice show that basal cells give rise to labeled basal, ciliated, and club cells, thus fulfilling the definition of progenitor cells.^22^, ^23^ Several studies suggest that basal cells from human trachea or bronchi will repopulate denuded tracheal xenografts or columnar epithelial cells *in vitro*.^24^, ^25^, ^26^, ^27^ However, there is no convincing evidence that a multipotent airway stem cell is capable of replenishing the epithelium in all intrapulmonary regions. Thus, tracheal, bronchiolar, and alveolar epithelia are likely maintained by regionally distinct progenitor cell lineages. Different Ad serotypes may transduce specific airway cells with varying efficacy, thus serotype selection is a potential strategy to direct tropism.

In this study, we compared 14 Ad vector types representing species C, B1, B2, D, and E for their ability to transduce the apical or basolateral surfaces of well-differentiated primary cultures of human airway epithelial cells. In addition, we examined the ability of the Ad types to transduce two *in vitro* models of basal cells: (1) conditionally reprogrammed tracheal basal cells and (2) primary basal cells from human donors. The results of this study have important implications for optimized gene delivery to airway cells.

## Results

**Figure 1:**
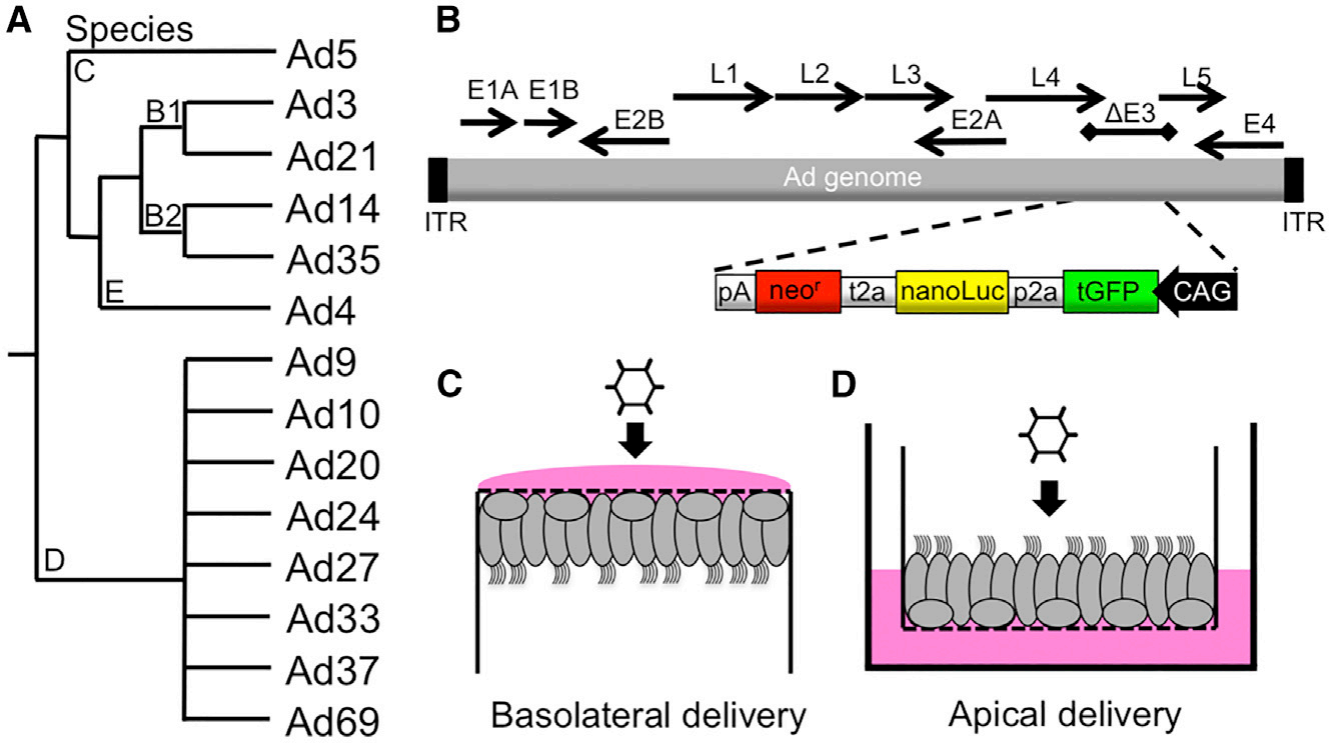
Ad Vectors and Delivery Protocol (A) The 14 Ad types from 4 species were screened for transduction properties in airway epithelial cells. (B) As shown schematically, each Ad type expressed tGFP, nanoluciferase (nanoLuc), and neomycin resistance (neo^r^) from the E3 genomic region. (C and D) Schematic (C) basolateral and (D) apical delivery protocols in polarized HAEs are shown. Vector was applied to the (C) basolateral surface by inverting the culture and applying the vector for ∼4 h. Following the incubation, the cultures were returned to the vertical position. (D) For apical delivery, vector was applied directly to the apical surface for 4 h, followed by washing. Following delivery, cultures were maintained at an air-liquid interface.

The 14 Ad types representing 4 species were chosen for this study. Here, the order of Ad types is based, in part, on phylogenic relationship and numerical order (Figure 1A). Ad5 serves as the basis of comparison and is listed first. As previously described,^3^ for each of the types tested, a transgene cassette containing turbo GFP (tGFP), nanoluciferase (nanoLuc), and neomycin resistance (neo^r^) is expressed from the E3 genomic region (Figure 1B).

### Transduction of Well-Differentiated Primary Cultures of Airway Epithelial Cells

**Figure 2:**
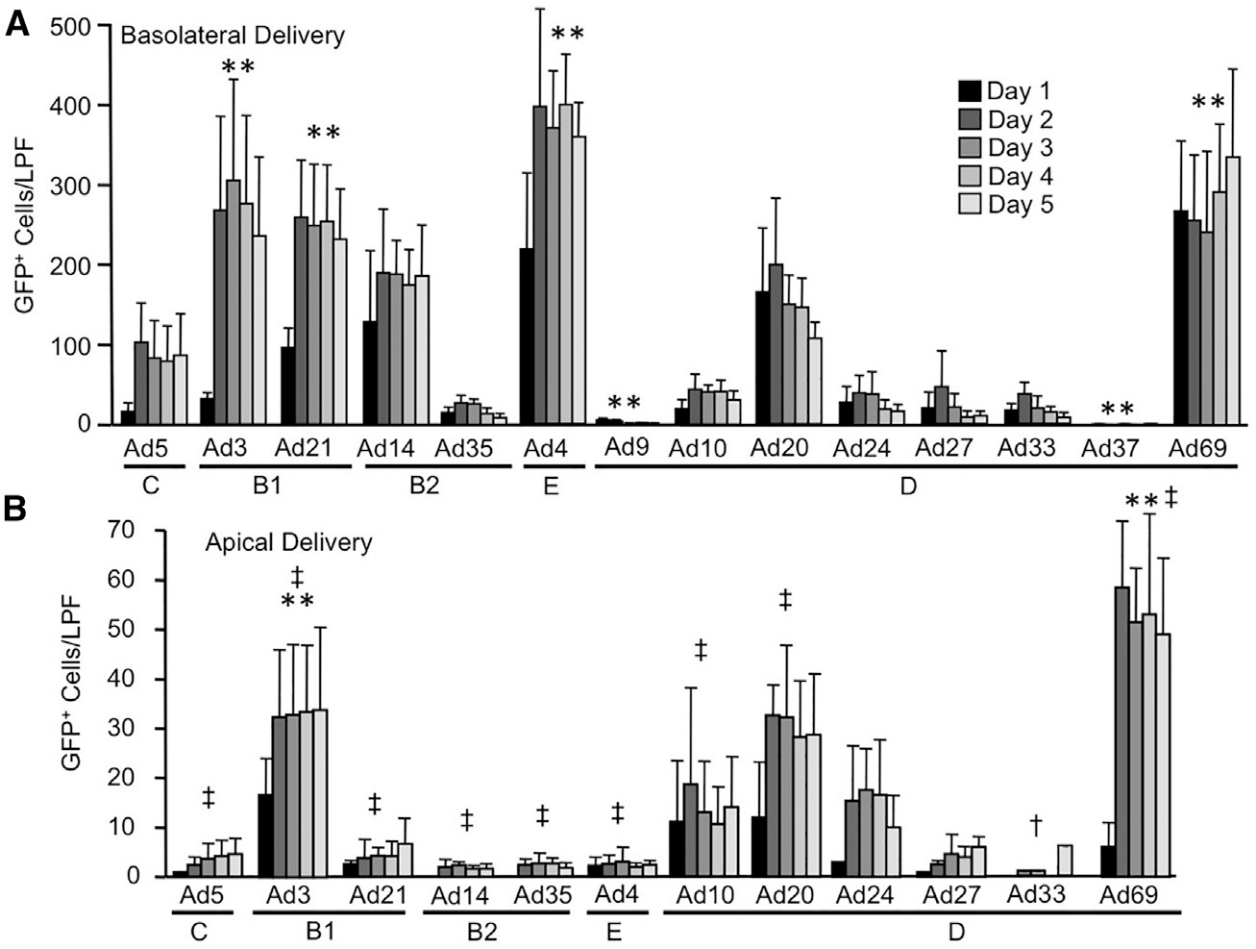
Time Course Quantification of Ad Transduction in HAEs (A) HAEs were transduced with the indicated Ad vectors from the basolateral surface at an MOI of 50. At days 1, 2, 3, 4, and 5 following vector delivery, GFP was documented using an inverted fluorescent microscope and a 10× objective. For each of 5 donors, 6 pictures were collected. The number of GFP-positive cells for each low-power field (LPF) was manually counted using ImageJ software. Statistical comparisons to Ad5: **p < 0.003, n = 5 donors. (B) HAEs were transduced with the indicated Ad vectors from the apical surface at an MOI of 50, and GFP^+^ cells were counted as in (A). Statistical significance tests were compared to Ad5. Statistical comparisons to Ad5: **p < 0.003. Statistical comparisons to basolateral application: ^†^p < 0.001, ^‡^p < 0.0001; n = 5 donors.

Our initial studies addressed three questions: (1) which Ad types efficiently transduce well-differentiated primary cultures of human airway epithelial cells (HAEs); (2) when does transgene expression reach a peak after cell transduction; and (3) are there Ad types that preferentially transduce the apical surface? As indicated, the Ad vectors were applied to the basolateral (Figure 1C) or apical (Figure 1D) surface of HAEs at an MOI of 50 for 4 h. Following basolateral (Figure 2A; Figure S1A) or apical delivery (Figure 2B), low-power (10×) images were collected daily for 5 days, and the numbers of GFP+ cells per low-power field (LPF) were quantified. In addition, after 5 days post-transduction, cell lysates were collected and nanoLuc expression was quantified.

Ad3, Ad21, Ad4, and Ad69 all resulted in significantly more GFP+ cells (Figure 2A) and greater nanoLuc expression (Figure S1B) than Ad5. Ad10, Ad14, Ad20, Ad24, Ad27, Ad33, and Ad35 were not statistically different than Ad5. Ad9 and Ad37 transduction resulted in significantly fewer GFP+ cells and lower nanoLuc expression. Interestingly, species B, E, and D each had members that resulted in better basolateral transduction of HAEs relative to Ad5. For all types tested, the number of GFP+ cells plateaued by 2 days post-transduction (Figure 2A). These data suggest that most Ad types transduce, traffic to the nucleus, and express their respective transgenes at a similar speed.

All serotypes but Ad24 and Ad27 resulted in significantly more GFP+ cells when applied to the basolateral surface as compared to the apical surface. For Ad24 and Ad27, the overall signal was weak, but there was also a trend for basolateral transduction preference. Ad3, Ad10, Ad20, Ad24, and Ad69 resulted in the best apical transductions, as determined by the number of GFP-positive cells (Figure 2B). No GFP+ cells were observed following the apical application of Ad9 or Ad37 (n = 2 donors, data not shown). In reference to our initial questions, (1) four Ad types transduced HAEs better than Ad5, (2) all Ad types tested reached maximal transgene expression by 2 days post-transduction, and (3) all Ad types preferentially transduced the basolateral surface of HAEs.

We next asked if similar trends of transduction efficiency would be observed following delivery to the airways of mice *in vivo*. The indicated Ad types were titer matched and delivered to mice airways via nasal instillation (Figure S2). At 2 days post-delivery, lungs were collected and homogenized. The nanoLuc expression was quantified by luciferase assay and normalized to tissue weight. In contrast to the observation in human cells, Ad5 delivery to mouse airways resulted in the highest level of nanoLuc expression. Of note, there is precedent for optimizing viral vectors in animal models, only to discover that transduction efficiencies in human cells are not reflected.^28^

**Figure 3:**
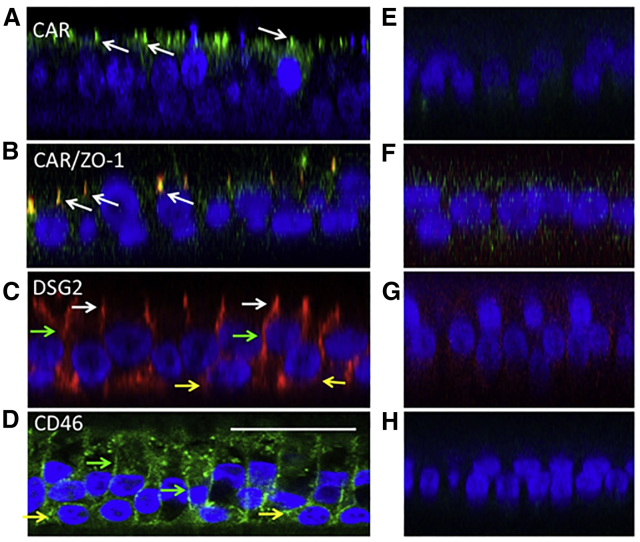
Ad Receptor Expression in HAEs (A–C) Immunohistochemistry was used to visualize (A and B) CAR (green), (C) DSG2 (red), and (D) CD46 (green). (B) CAR was co-stained with the tight junction marker ZO-1. White arrows indicate protein expression at the junctional complex between columnar epithelial cells. Green arrows indicate expression on the lateral surface of columnar epithelial cells. Yellow arrows indicate protein expression in basal cells. Nuclei were visualized with DAPI (blue). (E)–(H) are no-primary antibody negative controls to (A)–(D), respectively. Scale bar, 25 μm.

Species C, D, and E use CAR as the primary receptor. Similar to previous reports,^29^ we observed CAR expression localized to the tight junction region on the surface of HAEs (Figure 3A). To confirm tight junction localization, we co-labeled CAR with the tight junction marker ZO-1 (also known as TJP1 [tight junction protein 1]) (Figure 3B). The tight junctions define the border between the apical and basolateral membranes. DSG2 is the reported receptor for certain B species viruses, such as Ad3 and Ad14,^14^ and it is localized to the basolateral surface (Figure 3C). DSG2 expression was observed in the tight junctions of columnar epithelial cells (white arrows) and on basal cells (yellow arrows). CD46 is the reported receptor for Ad21 and Ad35 and has reported basolateral expression in polarized epithelial cells.^30^ Similar to DSG2, CD46 was observed on the lateral surfaces of columnar epithelial cells (green arrows) and on basal cells (yellow arrows) (Figure 3D). The corresponding no-primary controls are shown (Figures 3E–3H). Using flow cytometry, we determined that Ad5+ cells were 75% (±13.5%) CAR+, Ad3+ cells were 67% (±10.8%) DSG2+, and Ad21+ cells were 68% (±13.8%) CD46+. The expression patterns of CAR, DSG2, and CD46 on columnar epithelial cells were consistent with the observed preference for basolateral transduction.

### Transduction of Airway Basal Cells

**Figure 4:**
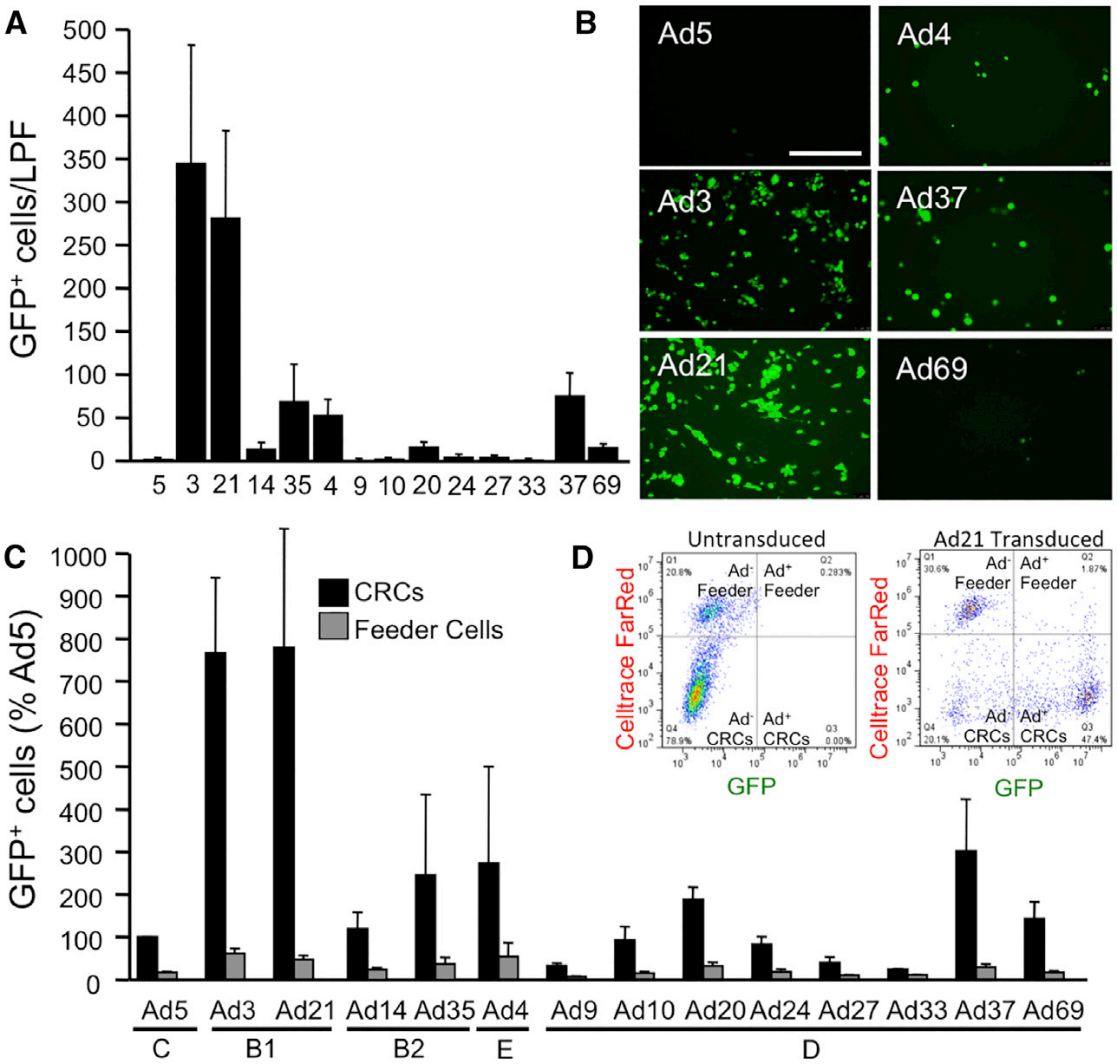
Ad Transduction of Conditionally Reprogrammed Cells (A) CRCs were transduced with the indicated Ad vectors at an MOI of 50. At 2 days post-delivery, GFP was documented using an inverted fluorescent microscope and a 10× objective. For each of 5 donors, 6 pictures were collected. The number of GFP-positive cells for each LPF was manually counted using ImageJ software. (B) Representative pictures of selected Ad types are shown. Scale bar, 500 μm. (C) Feeder cells were labeled with CellTrace Far Red prior to mixing with CRCs. The CRC and feeder cell co-culture was transduced with the indicated Ad vectors at an MOI of 50. At 2 days post-delivery, GFP+ and CellTrace+ cells were quantified using flow cytometry. (D) Sample scatterplots of untransduced and Ad21-transduced cells are shown.

We next asked if there were Ad types with better tropism for airway basal cells than Ad5. Using cells from HAEs, we conditionally reprogrammed cultures using Y-27632, a Rho-associated, coiled-coil-containing protein kinase (ROCK) inhibitor.^31^ This protocol yields basal airway progenitor cell populations from the heterogeneous cell population present in established cultures of HAEs. CRCs were co-cultured with morphologically distinct irradiated J2 feeder cells. As before, Ad types were applied to CRCs at an MOI of 50. Then 5 days later, low-power images were collected and GFP+ cells were quantified. Interestingly, we observed very few GFP+ cells following Ad5 transduction (Figures 4A and 4B). Whereas both species B1 vectors (Ad3 and Ad21) conferred the greatest number of GFP+ cell CRCs (Figures 4A and 4B), Ad35 (species B2), Ad4 (species E), and Ad37 (species D) also conferred significantly elevated levels of GFP+ cells as compared to Ad5.

In addition to manual cell counting, transduced cells were assessed by flow cytometry. For this experiment, irradiated J2 cells were labeled with CellTrace dye and washed prior to co-culturing with CRCs. At 5 days following Ad delivery, cells were sorted based on the presence of far red fluorescence (feeder cells) and/or GFP fluorescence (transduced cells). The results from counting by fluorescence-activated cell sorting (FACS) (Figure 4C) recapitulated the results from manual counting. Again, species B1 adenoviruses transduced CRCs with the greatest efficacy. Sample scatterplots are shown for untransduced CRCs and Ad21-transduced CRCs (Figure 4D).

**Figure 5:**
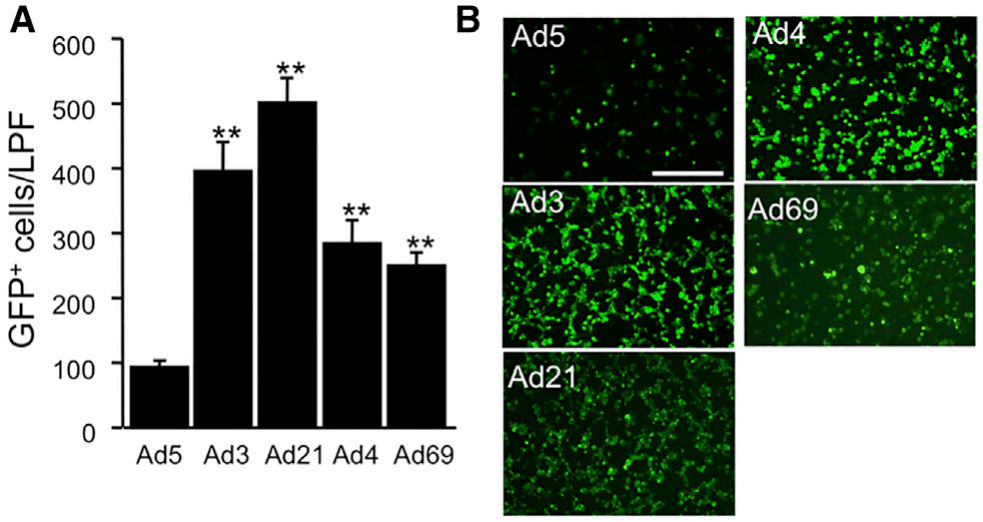
Ad Transduction of Primary Human Airway Basal Cells (A) Primary basal cells were transduced with the indicated Ad vectors at an MOI of 5. At 2 days post-delivery, GFP was documented using an inverted fluorescent microscope and a 20× objective. For each of 4 donors, 6 pictures were collected. The number of GFP-positive cells for each LPF was manually counted using ImageJ software. (B) Representative pictures of selected Ad types are shown. Scale bar, 50 μm. **p < 0.00001, n = 4 donors.

**Figure 6:**
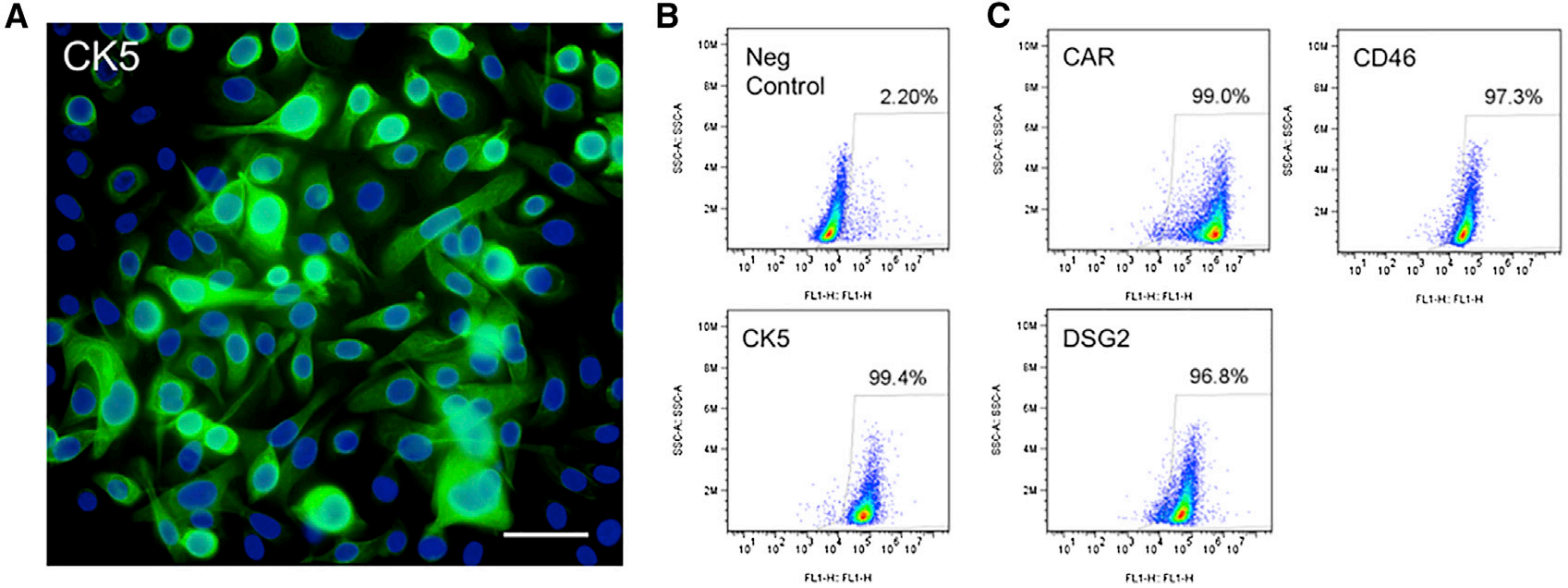
Basal Cell Markers and Ad Receptor Expression in Primary Human Airway Basal Cells (A and B) Immunohistochemistry (A) or flow cytometry (B) was used to visualize cytokeratin (CK5) in primary basal cells. Scale bar, 50 μm. (C) Flow cytometry was used to quantify CAR, DSG2, and CD46 in primary basal cells.

Primary cultures of bronchial basal cells were freshly isolated from human lung donor tissue, and they provide an alternative cell culture model to CRCs. Here we focused our attention on Ad5 and the 4 leading candidates (Ad3, Ad21, Ad4, and Ad69) for airway cell transduction. Consistent with CRCs, we observed that the species B1 adenoviruses transduced primary basal cells with the greatest efficiency (Figures 5A and 5B). Transduction levels of Ad3 and Ad21 reached efficiencies that were 4- to 5-fold over Ad5. Ad4 and Ad69 transduced primary basal cells 2- to 3-fold better than Ad5. The population of primary basal cells was confirmed to predominately (∼98%) express the basal cell marker CK5 (Figures 6A and 6B). In addition, CAR, CD46, and DSG2 were all abundantly expressed in primary basal cells (Figure 6C).

## Discussion

For gene therapy applications, adenovirus based vectors are attractive for multiple reasons. Ad vectors efficiently deliver DNA into the nuclei of dividing and nondividing target cells, can be amplified to high titers, are stable, withstand rigorous purification, and can be frozen for long-term storage. Ad2 and Ad5 are the most commonly used serotypes in the gene therapy field; however, they are unlikely to be the optimal serotypes for all human tissues of therapeutic interest. Vectors with improved transduction efficiencies would allow for reduced vector dosing and subsequent reduction in potential toxicity.

In this study, our goal was to identify adenovirus serotypes with improved tropism for HAEs. Ad5 is commonly used for many gene transfer applications; however, the adenovirus family has many members that vary greatly in their tissue tropism and pathologies. Based on availability and ease in production, we chose to contrast the airway cell tropism of Ad5 with 13 additional types. All types tested preferentially transduced the basolateral surface of HAEs over the apical surface. This result is consistent with the observed basolateral expression of the viral receptors. Of note, each species of adenovirus tested had at least one member that transduced HAEs better than Ad5. This suggests that primary receptor usage or abundance alone is insufficient to explain transduction efficiency. Receptor co-factors or post-entry barriers may also contribute to transduction efficiency. In addition to well-differentiated airway epithelial cells, we examined transduction efficiency in two *in vitro* models of airway basal cells. Conditionally reprogrammed cells and primary basal cells express basal cell markers as well as all 3 putative adenoviral receptors. Of interest, the members of species B1, Ad3 and Ad21, transduced both basal cell models significantly better than Ad5. Ad3 has been used in human clinical trials as an oncolytic vector,^32^ and it has a well-defined cell entry mechanism using DSG2.^14^

Previous efforts using chimeric fiber vectors suggest improved transduction of fibroblasts, dendritic cells, endothelial cells, and smooth muscle cells.^33^ Interestingly, Havenga and colleagues^33^ showed that the fibers from species B adenoviruses (e.g., Ad16, Ad35, and Ad50) were most effective at broadening the tropism range in a variety of cancer and primary cell lines. However, the one lung-derived cell line tested, A549, was best transduced by Ad5. Although Ad16 and Ad50 were not included in our study, our results were consistent with Ad5 outperforming Ad35 in epithelial cells isolated from human airways. In addition, our studies suggest that Ad5 is an appropriate choice for studies in mouse airways. Currently, the discrepancy between the results in mouse lungs *in vivo* and the primary cultures of human airway epithelia *in vitro* is unclear. Species specificity represents a challenge for bridging gene therapy preclinical studies and clinical trials. Indeed, gene therapy clinical trials for CF using adeno-associated virus serotype 2 (AAV2) provide a cautionary tale for a vector that efficiently transduced the airways of animal models but performed poorly in human airways.^28^, ^34^, ^35^

Ultimately, our goal is to incorporate these vector platform modifications into a clinical therapeutic for CF gene therapy. *CFTR* gene delivery using Ad2- and Ad5-based vectors represent flagship gene therapy phase 1 trials of the early 1990s. In total, there were 9 clinical trials for CF using Ad as the delivery vehicle (reviewed in^55^). With general agreement, these studies suggested that Ad-based vectors partially correct the Cl^−^ transport defect in CF airway epithelia; however, the effects were transient and inflammatory responses limited persistent phenotypic correction. One of the reasons Ad was selected as a gene transfer vector was because it is a respiratory virus and was assumed to have high transduction levels in the lung. Since the initial studies, considerable progress has been made describing interactions between Ad and its cellular receptors. For example, neither Ad2 nor Ad5 enters airway cells through the apical surface. In 1997, the cellular receptor for Ad2 and Ad5 was discovered to be CAR,^8^ and it is localized to the basolateral surface of airway cells.^9^ Species B receptors CD46 and DSG2 were discovered in 2003 and 2011, respectively.^12^, ^14^ A better understanding of Ad tropism and entry mechanisms may contribute to the identification of novel gene delivery reagents.

A single dose therapeutic that will correct the CF lung disease for the life of the patient would be optimal; therefore, genomic modification of progenitor cells is likely required. With current vector technologies, there are at least 2 strategies to use Ad-based vectors to achieve lifelong correction of the CF phenotype. The first strategy involves using Ad to deliver DNA transposons. Such hybrid non-viral and viral vectors combine the production and delivery efficiencies of an encapsidated viral vector with the persistent expression of a DNA transposon. Sleeping Beauty was the first transposon delivered by an Ad5-based vector, and it was shown to lead to persistent transgene expression *in vitro* and *in vivo*.^4^, ^36^ We recently reported persistent expression following delivery of the DNA transposon *piggyBac* to airway cells using Ad5.^2^, ^5^ Thus, hybrid vectors are tools that convert episomal vectors into vectors capable of genomic integration.

The second strategy involves gene editing. Gene repair strategies using zinc-finger nucleases (ZFNs), transcription activator-like effector nucleases (TALENs), or meganucleases have existed for over a decade. CRISPR/Cas9 has the advantages of efficient cutting, ease in design, and ease in generation. CRISPR technology is responsible for an explosion in the field of gene editing. Gene correction of *CFTR* mutations using CRISPR technology is currently receiving deserved attention from multiple laboratories. However, *in vivo* gene repair faces obstacles that extend beyond those associated with simple gene addition. For each *CFTR* mutation, the efficiency of cutting with different guide RNAs and the efficiency of homologous recombination with different repair templates would need to be assessed. In addition, the low efficiency of homologous recombination in quiescent cells, such as basal cells, is a global challenge for the field of gene editing.

Importantly, both gene addition and gene repair strategies rely on efficient *in vivo* gene delivery. For both gene addition and gene repair to be a single-dose therapeutic, progenitor cells will need to be corrected. There appear to be several epithelial cell types in the lung that provide these functions, which has led to controversy regarding which cells to target for CF gene therapy. Arguments can be made in support of the necessity of correcting basal cells^37^, ^38^ and non-ciliated columnar cells of the airways,^39^, ^40^, ^41^ submucosal glands (SMGs),^42^, ^43^, ^44^ club cells,^45^, ^46^ and alveolar type II cells^47^, ^48^ in the distal lung. Recent evidence suggests that ionocytes are important airway cell targets for correcting the CF phenotype.^49^, ^50^

Our data indicate that species B1 adenoviruses may be better choices than Ad5 for airway gene therapy. Ad3 and Ad21 transduced well-differentiated airway epithelial cells and airway basal cells more efficiently than Ad5. Additional Ad types could be screened for airway cell tropism. Within species B alone, Ad7, Ad11, Ad16, Ad34, Ad50, and Ad55 are all potential candidates to examine. Based on Centers for Disease Control and Prevention (CDC) summaries of voluntary Ad detections reported through the National Adenovirus Type Reporting System (NATRS),^51^ Ad21 may be less seroprevalent in the general population than other adenoviruses with similar improved airway tropism (i.e., Ad3 and Ad4). Gene therapy trials would likely exclude patients with pre-existing immunity to the vector serotype; as such, we speculate that Ad21- or Ad69-based vectors may be available to a broader pool of patients. Moving forward, the generation of novel Ad vectors with specific cellular tropisms could enhance delivery efficiency and lessen the minimum effective dose necessary for therapeutic benefits.

## Materials and Methods

### Vector Production and Titering

Recombinant adenoviral vectors expressing tGFP, nanoLuc, and neo^r^ were generated as previously described.^3^ The University of Iowa Viral Vector Core (https://medicine.uiowa.edu/vectorcore/) amplified the vectors in 293T cells. Vector particles were measured by spectrophotometry, and vector titers were determined by flow cytometry using GFP expression and limiting dilutions. Comparable titers ranging from 1 × 10^10^ to 5 × 10^10^ transducing units were generated for each vector type.

### Primary Cultures of HAEs

Primary cultures of HAEs were prepared from trachea and bronchi by enzymatic dispersion, using established methods.^52^ Briefly, epithelial cells were dissociated and seeded onto collagen-coated, semi-permeable membranes with a 0.4-μm pore size (Costar, Cambridge, MA, USA). Human airway epithelial cultures were maintained in Ultraser G (USG) media at 37°C and 5% CO_2_. Transwell inserts were placed into 24-well plastic cell culture plates (Costar, Cambridge, MA, USA). At 24 h after seeding, the mucosal medium was removed, and the cells were allowed to grow at the air-liquid interface as reported previously.^52^ Unless otherwise indicated, only well-differentiated cultures (>3 weeks old) were used in these studies. The presence of tight junctions was confirmed by measuring the transepithelial resistance using a volt-ohm meter (World Precision Instruments, Sarasota, FL, USA; resistance >500 Ω⋅cm^2^).

### Ethical Statement

Primary cultures of HAEs were prepared from discarded tissue, autopsy, or surgical specimens. All specimens used in this study were obtained from The University of Iowa *In Vitro* Models and Cell Culture Core Repository. We were not provided with any information that could be used to identify a subject. All studies involving human subjects received University of Iowa Institutional Review Board approval.

### Transduction of Primary Airway Epithelial Cells

To transduce airway epithelia with Ad vectors from the basolateral side, the transwell culture insert containing the airway epithelial culture was turned over, and virus was applied to the basolateral surface for 4 h in 100 μL serum-free medium. Following the 4-h incubation, the virus was removed, and the culture was turned upright and allowed to incubate at 37°C and 5% CO_2_, for the indicated time periods. For apical infection, Ad vectors were simply applied to the apical surface for 4 h in 100 μL serum-free medium. After the incubation, cells were rinsed with MEM three times to remove residual virus. At the indicated time points, 6 pictures/well were collected using an inverted fluorescent microscope and a 10× objective. GFP-positive cells were counted using ImageJ software. Cells were lysed, and nanoLuc levels were quantified by Nano-Glo Luciferase Assay System (N1110, Promega), using the manufacturer’s protocol.

### CRCs and Primary Basal Cells

CRCs were isolated from fully differentiated HAEs and reprogrammed as described previously.^53^ Briefly, cells were lifted from transwell cultures and maintained in F media in the presence of 10 μM Y-27632, a ROCK inhibitor, and low passages of irradiated fibroblast feeder cells (NIH 3T3-J2) at 37°C and 5% CO_2_. For primary basal cells, HAEs were isolated directly from donor trachea and bronchi as previously described.^52^ Cells were seeded onto collagen-coated tissue culture dishes, cultured in BronchiaLife Basal Medium supplemented with BronchiaLife LifeFactors (Lifeline Cells Techonology, Walkersville, MD, USA), and maintained at 37°C and 5% CO_2_.

### Flow Cytometry Staining

CRCs were mixed with CellTrace Far Red- (C34564, Invitrogen) labeled feeder cells. The CRC and feeder cell co-culture was transduced with Ad vectors at an MOI of 50. At 48 h post-delivery, cells were detached with ACCUMAX (SCR006, Millipore Sigma), washed with PBS, and fixed with 3.7% paraformaldehyde before being analyzed using a BD Accuri C6 flow cytometer. To quantify the cell surface receptor abundance, 10^6^ cells were incubated with rabbit polyclonal CAR1605p (a gift from Joseph Zabner), followed by Alexa Fluor488-conjugated anti-rabbit immunoglobulin G (IgG) antibody, fluorescein isothiocyanate (FITC)-conjugated mouse anti-human CD46 (Clone E 4.3, BD Biosciences), or Dsg2 antibody (AH12.2) Alexa Fluor488 (SC-80663AF488, Santa Cruz Biotechnology) on ice for 1 h. To verify CK5 expression, detached cells were permeabilized using the Cytofix/Cytoperm Fixation/Permeabilization Kit (554714, BD Biosciences), and they were incubated with anti-cytokeratin 5 antibody (MA5-17057, Thermo Fisher Scientific), followed by Alexa Fluor488 anti-mouse IgG antibody.

### Immunostaining

Fixed and permeabilized HAEs were incubated with anti-CAR (CARex75490, a gift from Joseph Zabner) followed by Alexa Fluor488 anti-rabbit IgG antibody, anti-dsg2 (human DSG2-biotinylated antibody; BAF947, R&D Systems) followed by donkey anti-goat IgG NorthernLights NL557-conjugated antibody (NL001, R&D Systems), or anti-CD46 (C-10) (SC-166159, Santa Cruz Biotechnology) followed by Alexa Fluor488 anti-mouse IgG antibody. Slides were counterstained and mounted with Vectashield containing DAPI (Vector Laboratories, Burlingame, CA, USA).

### *In Vivo* Delivery and Luminescence

All animal procedures were approved by the Institutional Animal Care and Use Committee (IACUC) and in accordance with NIH guidelines. All mice for this study were housed at The University of Iowa Animal Care Facilities. Female mice (6–8 weeks old) were transduced intranasally with each Ad serotype formulated 1:1 with 2% methylcellulose (50 μL total volume), as previously described.^5^, ^54^ At 48 h after viral instillation, mice were humanely euthanized by CO_2_ inhalation and lungs were collected. Lungs were weighed before being homogenized in tissue lysis buffer. nanoLuc expression was quantified using the nanoLuc substrate according to the manufacturer’s instructions (Promega, Madison, WI, USA).

### Statistics

All numerical data were presented as the mean ± SE. Statistical analyses were performed using GraphPad Prism software. Two-tailed, unpaired Student’s t tests were used to compare experimental groups. When necessary, Bonferroni corrections were performed to control for additional type 1 error for multiple analyses. F tests were performed in parallel to determine that variance was similar between groups. For nonparametric data, the Mann-Whitney U-test was used.

## Author Contributions

Conceptualization, P.L.S. and A.E.; Investigation, N.L., A.L.C., and W.Z.; Resources, W.Z. and A.E.; Writing, N.L., A.L.C., and P.L.S.; Visualization, P.L.S.; Funding Acquisition, P.L.S.

## Conflicts of Interest

The authors declare no competing interests.

## Supplemental Information

Document S1. Figures S1 and S2

Document S2. Article plus Supplemental Information
