# Supplementary material for: Enhanced Tropism of Species B1 Adenoviral-Based Vectors for Primary Human Airway Epithelial Cells
Source: Mol Ther Methods Clin Dev. 2019 Jul 12;14:228–36. doi: 10.1016/j.omtm.2019.07.001 (PMC6690641; doi:10.1016/j.omtm.2019.07.001)
Supplement: Document S1. Figures S1 and S2 [file mmc1.pdf]

OMTM, Volume *14*

Supplemental Information

Enhanced Tropism of Species B1 Adenoviral-Based Vectors for Primary Human Airway Epithelial Cells

Ni Li, Ashley L. Cooney, Wenli Zhang, Anja Ehrhardt, and Patrick L. Sinn

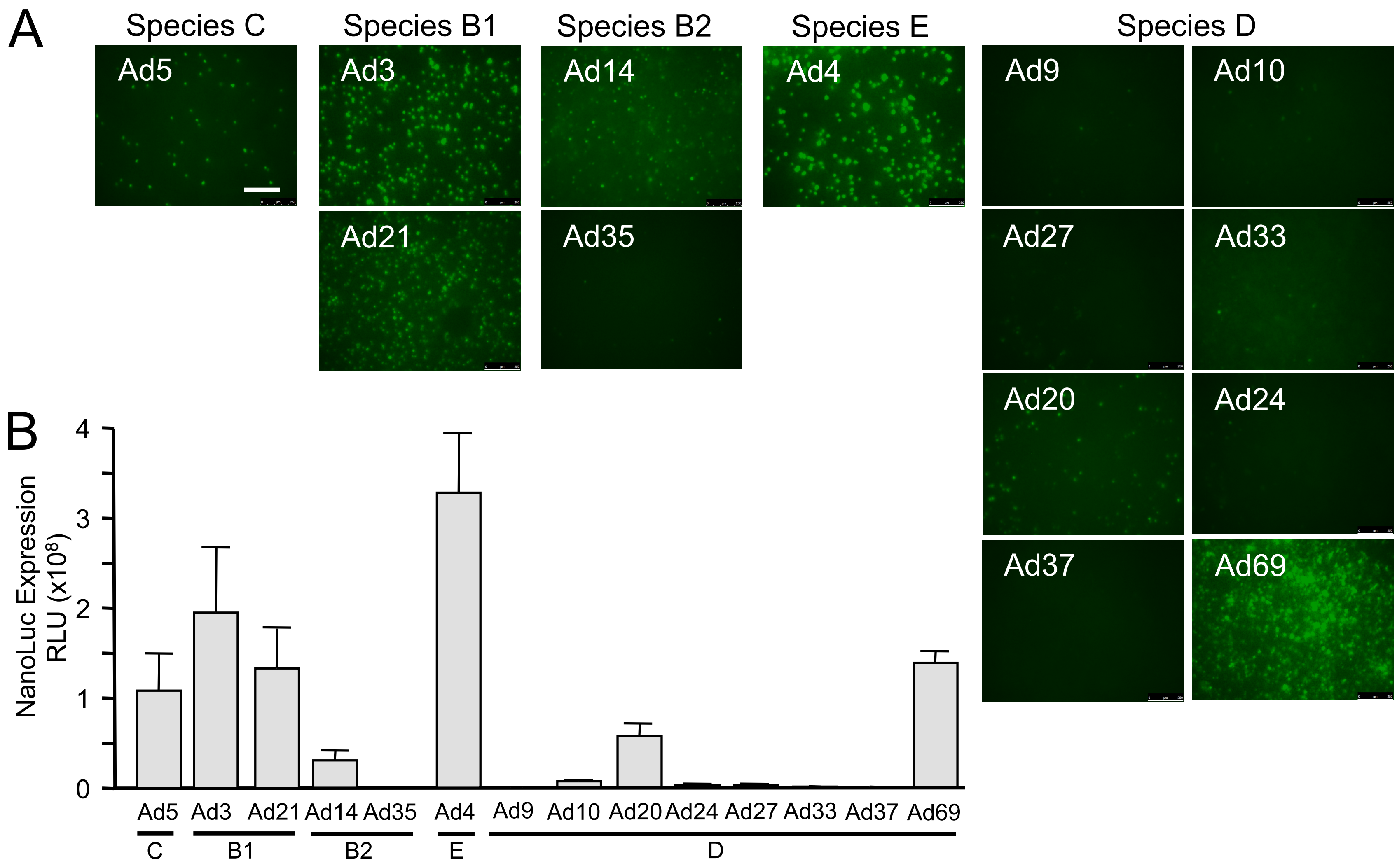

Supplemental Figure 1. Ad transduction of HAE. HAE were transduced with the indicated Ad vectors from the basolateral surface at an MOI of 50. A) Two days later, GFP was visualized using an inverted fluorescent microscope. Scale bar = 250 microns. Representative images from 5 donors are shown. At days 1, 2, 3, 4, and 5 following vector delivery, GFP was documented using an inverted fluorescent microscope and a 20X objective. For each of 5 donors, 6 pictures were collected. B) Following image collection at 5 days post-delivery, cell lysates were collected and nanoLuciferase levels (nanoLuc) were quantified using a commercially available luciferase assay (n = 5 donors).

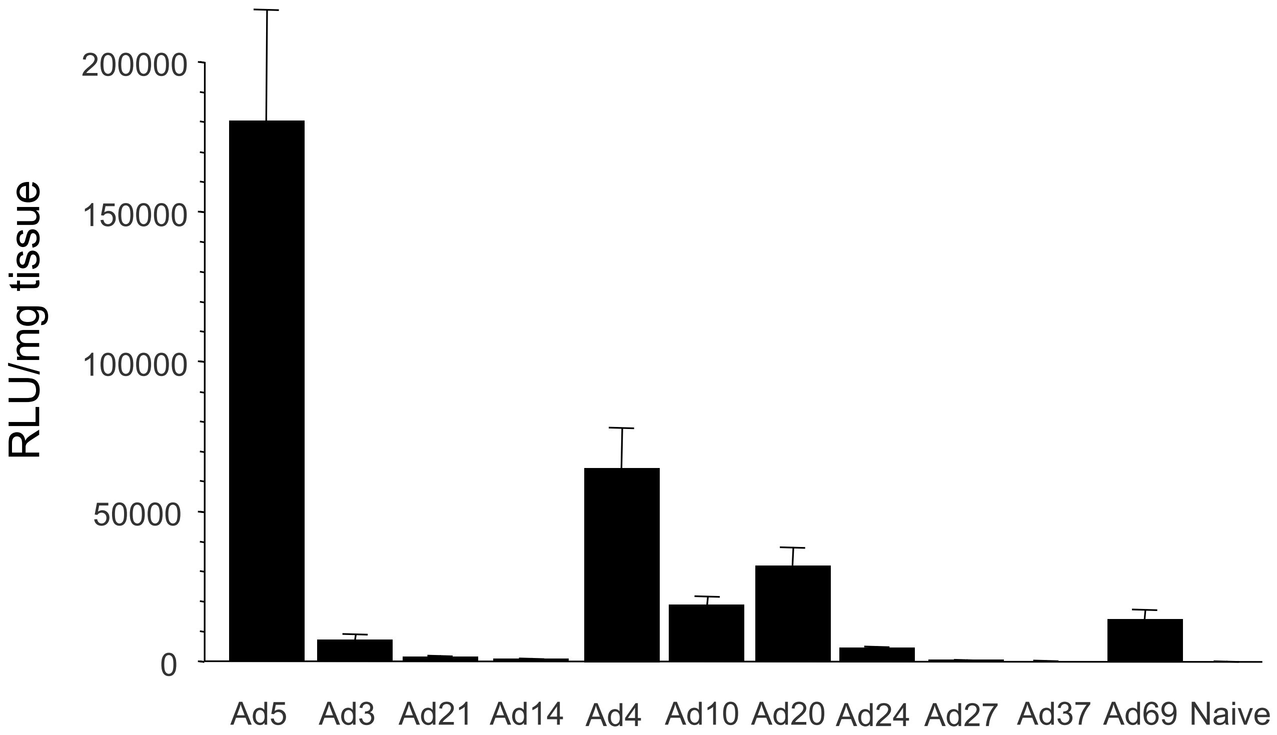

Supplemental Figure 2. Luciferase expression following Ad delivery to mouse airways. Indicated Ad serotypes were administered intranasally to 6-8 week old Balb/c mice. $2.5x10^7$ transducing units (TUs) were co-delivered with a 1:1 mixture of 2% methylcellulose in a total volume of 50 µl. 48 hours later, lungs were harvested and nanoluciferase expression was quantified using a luminometer. Luminescence relative light units (RLUs) were normalized to mass of lung tissue. N = 7.
